# Authorized Generics as a Manufacturer Response to the Medicaid Rebate Cap Removal

**DOI:** 10.1001/jamahealthforum.2026.0680

**Published:** 2026-05-08

**Authors:** Zeid S El-Kilani, Sanika Kulkarni, Joseph F. Levy

**Affiliations:** 1 School of Public Policy and Management, Heinz College of Information Systems and Public Policy, Carnegie Mellon University, Pittsburgh, Pennsylvania; 2 Department of Health Policy and Management, Johns Hopkins Bloomberg School of Public Health, Baltimore, Maryland

## Abstract

This cross-sectional study assesses the utilization of branded vs generic dapagliflozin and the financial implications of switching to generics under the Medicaid fee-for-service and managed care organization payment structures.

## Introduction

In 2024, the statutory cap on Medicaid drug rebates was eliminated.^1,2,3^ Under this change, manufacturers whose prices increased faster than inflation could face Medicaid rebate obligations that exceed gross sales to Medicaid, resulting in negative net revenue on those units.^4^ The policy intended to encourage price reductions; however, manufacturers may respond through alternative strategies that reduce rebate exposure and increase net revenue.^5^ One such strategy is shifting utilization from a branded product to an authorized generic (AG) product, which has lower rebates^5^ (eAppendix 1 in Supplement 1 explains Medicaid rebates). In this study, we examined this behavior with the branded dapagliflozin, Farxiga (AstraZeneca; eAppendix 3 in Supplement 1), a top-selling drug with no generic competition that had an AG launch in 2024, to assess how utilization shifted across Medicaid payment types following the removal of the Medicaid rebate cap.

## Methods

We conducted a retrospective analysis using publicly available Medicaid State Drug Utilization Data from 2021 to 2024. We identified quarterly utilization of branded and AG dapagliflozin and calculated the AG share of prescriptions. Utilization patterns were stratified by Medicaid payment structure: fee-for-service (FFS), managed care organizations (MCOs) with preferred drug lists (PDLs), and MCOs without PDLs (eTable 2 in eAppendix 2 in Supplement 1). In accordance with 45 CFR §46.104, this cross-sectional study was exempt from ethics review because publicly available aggregate data were used. We followed the STROBE reporting guideline.

To assess financial implications, we constructed simulations from the manufacturer and Medicaid program perspectives using observed utilization volumes and assumptions regarding statutory rebates and net pricing (eTable 1 in eAppendix 2 in Supplement 1). For the manufacturer’s perspective, 2023 utilization volumes were used as a counterfactual baseline to evaluate exposure to the 2024 rebate cap removal, independent of utilization changes. For the Medicaid perspective, analyses focused on 2024, the first year in which the policy change and AG uptake jointly affected program spending. Analysis was performed with Stata, version 19.5 Parallel Edition (StataCorp LLC).

## Results

Total utilization of dapagliflozin increased from 360 580 prescriptions in 2021 to 1 392 537 prescriptions in 2024 (Figure, A). Following the AG launch and rebate cap removal, 13.5% of utilization shifted from the branded to the AG product in 2024.

**Figure.**
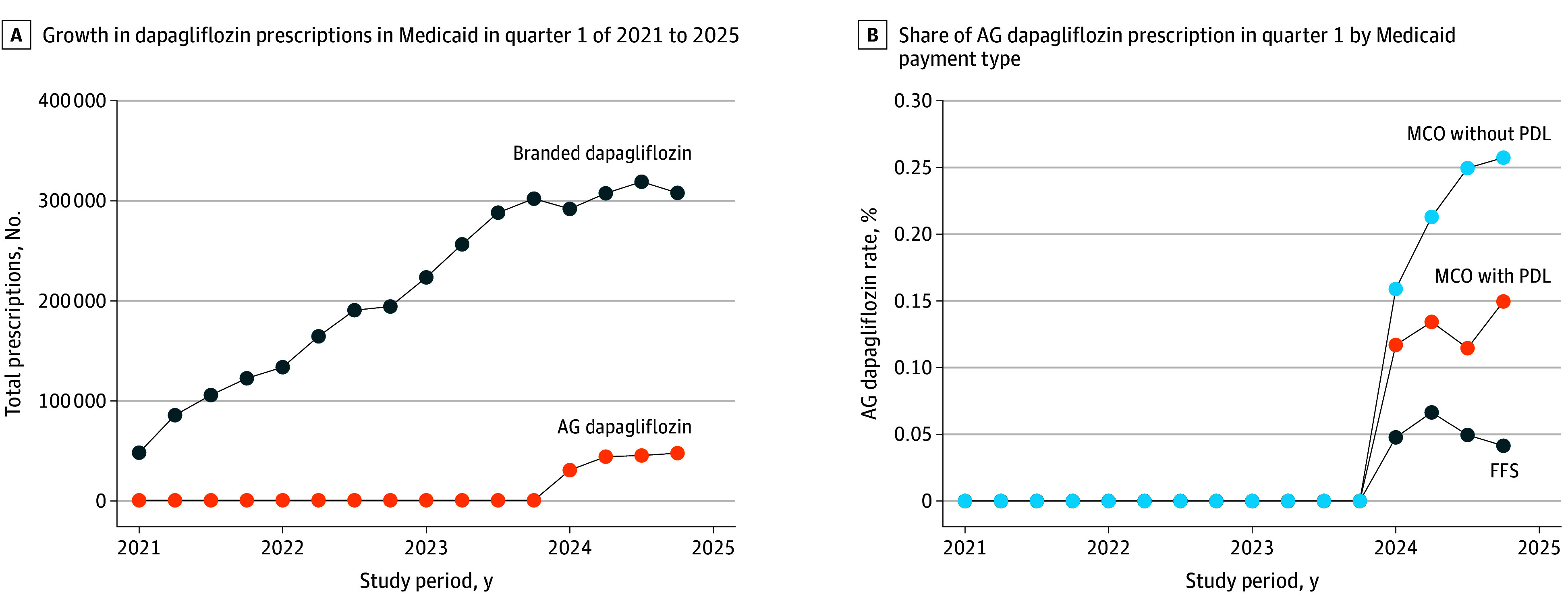
Line Graphs of Medicaid Prescriptions for Dapagliflozin and Share of Authorized Generic (AG) Dapagliflozin by Medicaid Payment Type Analysis was based on Medicaid State Drug Utilization Data. Managed care organization (MCO) programs were split based on state program responses to a 2024 Health Management Associates survey (eAppendix 2 in Supplement 1 provides more information). FFS indicates fee-for-service, and PDL indicates preferred drug list.

AG dapagliflozin uptake varied markedly by Medicaid payment type. By quarter 4 of 2024, MCOs without PDLs exhibited the highest AG penetration, reaching 25.7% of all dapagliflozin prescriptions. MCOs with PDLs showed intermediate uptake levels (15.0% in quarter 4 of 2024), while FFS Medicaid demonstrated substantially lower adoption, peaking at 6.6% in quarter 2 but declining to 4.1% by quarter 4 of 2024 (Figure, B).

These utilization shifts had large financial implications. Applying 2024 rebate rules to 2023 utilization volumes would have generated manufacturer losses of $33 623 407 (Table). Under observed 2024 utilization, including AG uptake, net losses were limited to $2 701 453. In contrast, under the counterfactual scenario with no AG, manufacturer losses would have been $40 777 610.

**Table.** Financial Implications of AG Dapagliflozin Adoption Under Alternative Medicaid Rebate Scenarios^a^

Year of utilization volume and rebate rules	Volume, No. of pills prescribed		Amount, $		
	Branded dapagliflozin	AG dapagliflozin	Gross revenue or spending	Rebates owed or received	Net revenue or spending
**Manufacturer perspective **					
2023 Volume, 2023 rules	43 188 606	NA	Revenue: 788 001 218	Owed: 788 001 218	NA
2023 Volume, 2024 rules	43 188 606	NA	Revenue: 788 001 218	Owed: 821 624 625	Revenue: −33 623 407
2024 Observed volume, 2024 rules	46 457 341	5 920 698	Revenue: 914 131 475	Owed: 916 832 929	Revenue: −2 701 453
2024 Volume, 2024 rules (no AG counterfactual)	52 378 039	NA	Revenue: 955 667 764	Owed: 996 445 374	Revenue: −40 777 610
**Medicaid perspective**					
FFS					
2024 Observed volume, 2024 rules	23 683 248	1 066 498	Spending: 453 154 643	Received: 456 501 169	Spending: −3 346 527
2024 Volume, 2024 rules (no AG counterfactual)	24 749 746	NA	Spending: 460 789 286	Received: 470 841 795	Spending: −10 052 509
MCOs with PDLs					
2024 Observed volume, 2024 rules	11 539 448	1 758 750	Spending: 234 994 847	Received: 229 337 364	Spending: 5 657 483
2024 Volume, 2024 rules (no AG counterfactual)	13 298 198	NA	Spending: 247 585 052	Received: 252 986 330	Spending: −5 401 278
MCOs without PDLs					
2024 Observed volume, 2024 rules	11 234 645	3 095 450	Spending: 244 637 730	Received: 230 994 395	Spending: 13 643 334
2024 Volume, 2024 rules (no AG counterfactual)	14 330 095	NA	Spending: 266 796 849	Received: 272 617 248	Spending: −5 820 399

Abbreviations: AG, authorized generic; FFS, fee-for-service; MCO, managed care organization; NA, not applicable; PDL, preferred drug list.

^a^ Analysis based on National Average Drug Acquisition Cost (NADAC) files, US Bureau of Labor Statistics Consumer Price Index for All Urban Consumers, Medicare Maximum Fair Price, estimates of net prices, and Medicaid State Drug Utilization Data. The assumed average manufacturer price (AMP) was 98% of NADAC and best price was 95% of the maximum fair price. Total rebates per unit for brand-name and authorized generic drugs were calculated using these assumptions. Gross spending per unit was calculated based on the assumption that Medicaid reimbursed pharmacies NADAC and manufacturers received the estimated AMP in gross revenue. eAppendix 2 in Supplement 1 provides an explanation of the calculations.

In FFS Medicaid, AG uptake modestly reduced net rebate collections but resulted in higher net spending compared with the no-AG counterfactual (−$3 346 527 vs −$10 052 509). Among MCOs with PDLs, AG adoption partially offset increased spending associated with higher utilization. MCOs without PDLs experienced the largest product-mix shifts and changes in gross spending and rebate flows, consistent with weaker formulary control (Table).

## Discussion

Removing the Medicaid drug rebate cap created strong incentives for manufacturers to reduce their exposure to inflation-based rebates. For dapagliflozin, this incentive was primarily achieved by redirecting utilization toward an AG product rather than through price reductions. AG uptake varied systematically across payment structures, being highest among MCOs without PDLs, intermediate among MCOs with PDLs, and lowest for FFS.

These differences are consistent with misaligned financial incentives in managed care. MCOs are typically paid based on gross drug spending rather than net-of-rebate costs because statutory rebates accrue to state Medicaid programs rather than to plans.^2^ Consequently, MCOs may have limited incentives to prioritize products that increase rebate collections or reduce net Medicaid spending. In contrast, FFS Medicaid, which directly benefits from rebates, exhibited substantially lower AG adoption.

Study limitations include our imprecise rebate estimates, focus on a single drug, and categorization of states based on adoption of uniform PDL policies rather than their PDL. Direct rebate data would improve the precision of these estimates. Classifying states by adoption of uniform PDL policies does not guarantee that AG dapagliflozin was excluded from those formularies.

Overall, these findings suggest that payment structure and rebate pass-through play a central role in shaping manufacturer strategy and program-level financial outcomes following the rebate cap removal. Policymakers should consider options that ensure Medicaid programs minimize costs and include policies to control MCO formularies, such as uniform PDLs, incentives to MCOs for using highly rebated products, or federal changes to the rebate program that hold manufacturers accountable for AG strategies reducing rebate liability.

## Notes

Supplement 1. **eAppendix 1.** Background On Medicaid Drug Rebate Program (MDRP) **eAppendix 2.** Methods **eAppendix 3.** Use of Farxiga as a Case Study
eReferences

Supplement 2.
Data Sharing Statement
