# Supplementary material for: Authorized Generics as a Manufacturer Response to the Medicaid Rebate Cap Removal
Source: JAMA Health Forum. 2026 May 8;7(5):e260680. doi: 10.1001/jamahealthforum.2026.0680 (PMC13156780; doi:10.1001/jamahealthforum.2026.0680)
Supplement: Supplement 1. — eAppendix 1. Background On Medicaid Drug Rebate Program (MDRP) eAppendix 2. Methods eAppendix 3. Use of Farxiga as a Case Study eReferences [file jamahealthforum-e260680-s001.pdf]

Supplemental Online Content

El-Kilani ZS, Kulkarni S, Levy JF. Authorized generics as a manufacturer response to the Medicaid rebate cap removal. *JAMA Health Forum*. 2026;7(5):e260680.
doi:10.1001/jamahealthforum.2026.0680

eAppendix 1. Background On Medicaid Drug Rebate Program (MDRP)

eAppendix 2. Methods

eAppendix 3. Use of Farxiga as a Case Study

eReferences

This supplementary material has been provided by the authors to give readers additional details about their work.

© 2026 El-Kilani ZS et al. *JAMA Health Forum*

eAppendix 1. Background On Medicaid Drug Rebate Program (MDRP)

1. MDRP Purpose

The Medical Drug Rebate Program (MDRP) was designed to ensure access to and low-cost drugs for Medicaid beneficiaries.[1] To achieve this, the program statutorily requires Medicaid to pay the best price available in the commercial market (Best Price Rule); protect the program from price hikes exceeding inflation (the Inflation Penalty); and institute mandatory rebates and pricing transparency requirements to help states manage drug spending more effectively.[2] Over time, policy changes have expanded MDRP rebate requirements, increasing state and federal savings while raising manufacturer rebate obligations. Medicaid continues to pay the lowest average net prices across all market segments and federal programs.[3]

2. Stakeholders

Manufacturers

Participation in MDRP is mandatory for any manufacturer seeking Medicaid coverage for its outpatient drugs. Currently, all 50 states and DC participate in the MDRP, and 780 manufacturers have signed National Drug Rebate Agreements (NDRAs) with the Secretary of the Department of Health and Human Services (HHS).[4] Under this agreement, manufacturers must report product and pricing data monthly and quarterly on all their covered outpatient drugs (CODs) to CMS's Medicaid Drug Programs (MDP) system. They must also pay federal statutory and supplemental rebates on each covered, dispensed drug where applicable.[4,5] In exchange, Medicaid must cover nearly all FDA-approved drugs from the manufacturer, thus creating an open formulary. However, Medicaid plans can restrict access to drugs through the use of prior authorization, creating Preferred Drug Lists (PDLs).

Manufacturers can negotiate supplemental rebates with states and managed care organizations (MCOs) to be placed on their Preferred Drug List (PDL), a list of drugs providers are directed to prescribe that have limited to no utilization controls (prior authorization, step therapy, quantity limit, etc.), effectively driving sales towards that product. Not all states have PDLs, and some have PDLs for only certain classes of drugs.

CMS

CMS manages NDRAs, pricing, and product data through the MDP, calculates unit rebate amounts (URAs) for every National Drug Code (NDC), and facilitates rebate reconciliation between manufacturers and the state quarterly, and determines the federal share of each state's rebates through the Federal Medical Assistance Percentage (FMAP). In FY 2024, the FMAPs ranged from 50% to 77.27%.[6]

State Medicaid Agencies

State Medicaid agencies administer their pharmacy benefit through their fee-for-service program (FFS) or MCOs. States submit drug utilization data to manufacturers for rebate invoicing. States collect the URA but can also negotiate supplemental rebates from the

manufacturers in exchange for placement on their PDL, as explained earlier. The PDL can also be based on differences in statutory rebates and differences in clinical benefits. The rebate is shared between the state and the federal government.

Managed Care Organization (MCOs)

Some state agencies run their Medicaid Program through private managed-care vendors called MCOs. In such states, state Medicaid agencies pay MCOs a flat per-enrollee monthly fee based on the medical conditions of the enrollee. These per-enrollee rates are calculated yearly. The MCOs have the incentives to manage the care of enrollees in ways to reduce costs, because they pocket the difference between the payment and actual cost before the rates are adjusted the following year. We explore more of the consequences of these financial incentives in the methodology supplement below.

If a state contracts MCOs, it will generally either 'carve-in' or 'carve-out' its pharmacy benefit. If the pharmacy benefit is 'carved in,' the MCO will administer it; if it is 'carved out,' the FFS programs will administer it. When the benefit is carved in, the MCO must follow the state PDL if there is one and face financial and legal consequences if they do not. The MCO receives no portion of the rebates. In the case there is no state requirement to follow the state PDL, MCOs can negotiate supplementary rebates with the manufacturer for placement on the MCO's formulary. The status of each state's Medicaid program in 2024 is included below in the methodology supplement.

3B. Supplemental Rebates

States can negotiate supplemental rebates on top of MDRP's URA between therapeutically equivalent drugs to further reduce their prescription drug expenses. Supplemental rebates vary by state in how they are calculated.[7] Supplemental rebates are also shared between the state and federal government.

In exchange for the rebate, the state will put the manufacturer's drug on the state's Preferred Drug List (PDL). Drugs on the PDL also face little to no prior authorization, step therapy, quantity limits, or other utilization controls. Drugs not on the PDL face these controls. States either have no PDLs or uniform PDLs for some or all drug classes. PBMs must implement and providers must prescribe strictly from the PDL or face penalties.

3C. Recent Policy Changes

2010 Affordable Care Act

The ACA affected MDRP in two ways. First, the ACA increased the minimum AMP-based rebate from 15.1% to 23.1% for brand-name drugs and from 11% to 13% for generics. Second, the ACA expanded the MDRP rebate requirement to outpatient drugs purchased by patients in MCOs.[8] Previously, only drugs for FFS beneficiaries were covered. These two changes increased the URA owed per drug dispensed and increased the covered number of drugs dispensed,

© 2026 El-Kilani ZS et al. *JAMA Health Forum*

increasing the overall rebate amounts owed by the manufacturers and further generating greater state and federal savings.

2019 Change in Calculation of AMP

In September of 2019, President Biden signed Pub. L. 116-59, the Continuing Appropriations Act (2020) and Health Extenders Act (2019), which required manufacturers to calculate the AMP of brands and authorized generics separately rather than together.[9] Before the acts, authorized generics could lower the overall value of the AMP, thereby lowering the base rebate and inflation penalty of the URA that manufacturers were required to pay for brand-name drugs. This meant manufacturers were able to retain more revenue. The act intended to fairly calculate AMPs to reflect price variation amongst different drug types. However, the consequence of this action is that authorized generics and brands could face dramatically different rebate obligations. Since authorized generics have lower AMPs, they face lower base rebates and inflation rebates than the brand product. CMS clarified in guidance that even with these AMP changes, a single best price existed for the brand and authorized generic.[10] The implication is that for drugs with large inflation rebates, manufacturers could increase net revenues if a Medicaid patient receives the authorized generic rather than the brand version of the product.

2024 Rebate Cap Removal

The 2021 American Rescue Plan (ARP) Act made a major policy change to MDRP: it removed the rebate cap that stated the URA could not exceed the AMP. Prior to this change, even if the URA exceeded the AMP, the manufacturer only needed to pay the AMP in MDRP rebates, rendering the inflation penalty ineffective.

With the cap removal, enacted in January of 2024, the URA could be greater than the AMP. The manufacturer could have to pay Medicaid more than the drug's list price to have its drug covered under MDRP. The intent of the policy was to discourage manufacturers from increasing drug prices above inflation and to launch new drugs at lower prices to limit loss of revenue. Levy et al. (2024), however, observed manufacturers discontinue their branded product in favor of an authorized generic. Recall, the brand-name drug has a base rebate that is 23.1% of the AMP, while the generic's is only 13% of the AMP, reducing their loss to Medicaid. The OIG is also expected to issue two studies exploring the "Manufacturer responses to the Medicaid Drug Rebate Cap Removal" in 2026.[11]

© 2026 El-Kilani ZS et al. *JAMA Health Forum*

eAppendix 2. Methods

Calculations

To assess the impact of the launch of the authorized generic, we needed to estimate the potential rebates received by the Medicaid program, the net price received by manufacturers, and the potential gross spending on the ingredient costs paid for pharmacies.

To estimate the potential rebates, we first estimated the AMP and Best Price for the brand drug and the authorized generic. To estimate AMP, we used the publicly available National Average Drug Acquisition Cost (NADAC).[12] AMP and NADAC represent transactions at different levels of the supply chain. AMP represents the average price manufacturers receive when selling to wholesalers. Meanwhile, NADAC presents the average prices pharmacies pay for the drug from wholesalers. The difference between the two should be the wholesaler's markup. We assumed that AMP was 98% of NADAC to approximate a 2% markup. Prior research suggests that brand drug wholesaler markups range from 1% to 4%.[13] We conduct sensitivity analyses, changing this assumption about markups. We assumed the markup is similar for the authorized generic because, although generally wholesaler markups are larger for generics, that is due to competition among generics. In this case, the authorized generic is the only generic.

To estimate Best Price, we used the negotiated Medicare Maximum Fair Price[14] and assumed Best Price is 95% of this maximum fair price. We make this assumption because prior research suggests that the MFP is close to the average Part D rebate.[15] Best Price instead reflects the lowest net price among commercial payers. We assumed the commercial average would be similar to the Part D average, and the best price would be slightly below the average. We conduct sensitivity analyses on this assumption. With these assumptions in mind, we made several calculations.

First, we needed to calculate the base rebate for the brand and authorized generic. The key question is whether the base rebate is based on the statutory minimum or the best price. CMS released guidance after the 2019 change indicating that both the brand and the authorized generic share a single best price. In both cases, the difference between the AMP and the Best Price results in a larger rebate than the statutory rebate would be. Therefore, the base rebate was calculated as:

$$Base\ Rebate = Best\ Price - AMP$$

Where the AMP differs between the brand and the authorized generic, the implication is that although they have the same Best Price, the lower AMP results in a lower Base Rebate.

Next, we calculated the potential inflation rebate. To calculate the inflation rebate, we needed to determine the AMP at launch and in 2024. We found the first available NADAC in the 2014 NADAC file and applied the same procedures to get to AMP. We then used the CPI-U for March

2014 (the month preceding the first NADAC) and the CPI-U for December 2023 (the month before the January 2024 NADAC). We inflated the 2014 AMP to 2024. The difference between the 2024 AMP and the inflated 2014 AMP represented the inflation rebate for the brand drug. For the authorized generic, we did not include any inflation rebate. It was unclear from CMS guidance whether the inflation rebate applies to the authorized generic launch AMP; if so, there would be no inflation rebate because it just launched. Alternatively, the inflation rebate could have been based on the inflated launch brand AMP. In this case, there still would be no inflation rebate because the authorized generic AMP was less than the inflation-adjusted brand AMP.

The total rebate was calculated to be the sum of the base rebate and the inflation rebate. For situations where we assumed the rebate cap was not lifted, we capped the rebate at AMP.

Finally, we assumed the manufacturer did not give any supplemental rebates for the drug. We made the assumption based on the results of our analysis. These assumptions suggested that before the cap removal, potential rebates triggered the cap. Meanwhile, after the cap was removed, the manufacturer paid even more rebates. We assumed the manufacturer would not give rebates if they were making zero or a negative amount on the drug. The manufacturer may be giving rebates to MCO on the authorized generic, though. If that is the case, MCOs would have saved more money in 2024, and the brand manufacturer would have made less money.

Based on these assumptions, on a per-pill basis, we determined that manufacturers would receive

$$Manufacter\ Net\ Revenue = AMP - Best\ Price\ Rebate - Inflation\ Rebate$$

Where each of the three differs between brands and authorized generics. The difference between the best price and the AMP is the same for the brand and the authorized generics. As a result, manufacturer savings from launching authorized generics come from reduced inflation rebates.

Since we assume MCOs do not receive supplemental rebates, the only payments from the MCOs are to pharmacies.

$$MCO\ Cost = Payment\ to\ Pharmacies$$

We assume the payment to pharmacies for ingredient costs is based on the NADAC. Therefore, MCOs save the difference in NADAC between the brand and the authorized generics when using an authorized generic.

In the case of MCO Plans, state Medicaid programs only need to consider the rebate received.

$$Medicaid\ Rebates\ Recieved = Best\ Price\ Rebate + Inflation\ Rebate$$

In this case, the state Medicaid program receives smaller rebates because both the best-price rebate and the inflation rebate are lower for authorized generics. This rebate is shared with the federal government. We did not attempt to separate out the rebates accruing to states compared to the federal government because it differs by state.

© 2026 El-Kilani ZS et al. *JAMA Health Forum*

For FFS Medicaid Programs, it is a combination of the two effects for MCOs and the state program: the lower rebates are partially offset by lower rebates to pharmacies.

$$Net\ Cost\ for\ FFS\ Medicaid = Payment\ to\ Pharmacies - Best\ Price\ Rebate - Inflation\ rebate$$

In eTable 1 below, we lay out the calculations above

eTable 1: Per Pill Calculations Table

	5 MG	10 MG
AMP as a percent of NADAC	98%	98%
Best Price as a Percent of MFP	95%	95%
Brand AMP and Inflation Rebate Calculations		
Launch NADAC	$9.29	$9.30
Estimated AMP Launch	$9.10	$9.12
Inflation-Adjusted Launch AMP	$11.81	$11.83
2024 NADAC – Brand	$18.62	$18.62
Estimated 2024 Brand AMP	$18.25	$18.25
Inflation Rebate	$6.43	$6.41
Authorized Generic AMP Calculations		
Authorized Generic NADAC	$11.46	$11.78
Authorized Generic AMP	$11.23	$11.54
Best Price Rebate Calculations		
MFP	$5.95	$5.95
Estimated Best Price (applies to both Brand and AG)	$5.65	$5.65
Best Price Rebate – Brand	$12.59	$12.59
Best Price Rebate – Generic	$5.58	$5.89
Total Rebates		
Total Rebate Brand with Cap Removal	$19.02	$19.00
Total Rebate Generic with Cap Removal	$5.58	$5.89
Total Rebates Brand without Cap Removal	$18.25	$18.25
Total Rebates Generic without Cap Removal	$5.58	$5.89
Impact on Manufacturers		
Gross Revenue Manufacturer Brand (AMP)	$18.25	$18.25
Net Revenue Manufacturer Brand with Cap Removal	-$0.78	-$0.76

© 2026 El-Kilani ZS et al. *JAMA Health Forum*

Net Revenue Manufacturer Brand without Cap Removal	$0	$ 0
Gross Revenue Manufacturer Generic (AMP)	$11.23	$11.54
Net Revenue Manufacturer Generic with Cap Removal	$5.65	$5.65
Net Revenue Manufacturer Generic without Cap Removal	$5.65	$5.65
Impact on Medicaid FFS		
Gross Brand Drug Spend (NADAC)	$ 18.62	$ 18.62
Net Brand Drug Spend with Cap Removal	- $0.41	- $0.39
Net Brand Drug Spend without Cap Removal	$0.37	$0.37
Gross Generic Drug Spend (NADAC)	$ 11.46	$ 11.78
Net Generic Drug Spend with Cap Removal	$5.88	$5.89
Net Generic Drug Spend without Cap Removal	$5.88	$5.89
Impact on Medicaid with MCOs		
Gross Brand Drug Spend (NADAC) Paid by MCOs	$ 18.62	$ 18.62
Gross Generic Drug Spend (NADAC) Paid by MCOs	$ 11.46	$ 11.78
Total Brand Rebates with Cap Removal Received by Medicaid	$ 19.02	$ 19.00
Total Generic Rebates with Cap Removal Received by Medicaid	$5.58	$5.89
Total Brand Rebates without Cap Removal Received by Medicaid	$ 18.25	$ 18.25
Total Generic Rebates without Cap Removal Received by Medicaid	$5.58	$5.89

Categorizing Each State's Medicaid Program

To categorize each state's Medicaid program, we relied on a survey conducted by Health Management Associates.[16] Health Management Associates is a healthcare management consulting firm. For several years, they have surveyed state Medicaid agencies to understand the policy differences between states. We used the 2024 survey for this analysis. One question on the survey asks whether the state program requires MCOs to comply with a UPL. If MCOs managed the pharmacy benefit in the state, the state could indicate if they required a uniform PDL for all classes, a uniform PDL for some classes, or did not require a uniform PDL. We categorized only states that require a uniform PDL for all classes as the PDL states. To validate this classification, we compared information in the report to information in contracts between states and MCOs. States were classified as follows in eTable 2.

© 2026 El-Kilani ZS et al. *JAMA Health Forum*

eTable 2. Categorization of State Medicaid Programs

Category	N	States
MCOs with Preferred Drug Lists (PDLs)	23	AZ, CO, DC, FL, GA, HI, IA, KS, MD, MI, MS, NE, NH, NJ, NM, NV, OK, OR, RI, SC, UT, VA, WA
MCOs without Preferred Drug Lists (No PDLs)	11	AR, DE, IL, IN, KY, LA, MA, MN, NC, PA, TX
Fee-For-Service Only	17	AK, AL, CA, CT, ID, ME, MO, MT, ND, NY, OH, SD, TN, VT, WI, WV, WY
Note: All states (except Kansas and Nebraska) had some FFS utilization.		

Our classification has several notable limitations. States that require a uniform PDL for some classes could require a PDL for SGLT2 inhibitors, the class in which dapagliflozin belongs. As such, they may look more like states with uniform PDLs. Second, states that require uniform PDLs may not exclude the authorized generic dapagliflozin, making them look more like non-PDL states. Carefully scrutinizing the PDL development of each state was beyond the scope of this research letter.

© 2026 El-Kilani ZS et al. *JAMA Health Forum*

eAppendix 3. Use of Farxiga as a Case Study

In selecting Farxiga as a case study, we conducted an analysis examining the number of drugs that launched authorized generics after the 2019 change (effective October 1, 2019), through 2025. We excluded drugs that launched authorized generics after generic entry, focusing on drugs that could use authorized generics as a way to minimize rebate obligations. We find Farxiga particularly compelling because of the timing of the authorized generic launch (January 2024). The majority of the drugs in this group launched near the 2024 change (late 2023) or much later. Farxiga made an interesting example because the entry provided us with a way to compare full years of data.

eReferences

1. Rep. Panetta L [D C 16. Text - H.R.5835 - 101st Congress (1989-1990): Omnibus Budget Reconciliation Act of 1990. November 5, 1990. Accessed January 13, 2026. https://www.congress.gov/bill/101st-congress/house-bill/5835/text

2. Dolan R. Understanding the Medicaid Prescription Drug Rebate Program. KFF. November 12, 2019. Accessed January 13, 2026. https://www.kff.org/medicaid/understanding-the-medicaid-prescription-drug-rebate-program/

3. *A Comparison of Brand-Name Drug Prices Among Selected Federal Programs*. Congressional Budget Office; 2021. Accessed January 13, 2026. https://www.cbo.gov/system/files/2021-02/56978-Drug-Prices.pdf

4. Medicaid Drug Rebate Program (MDRP) | Medicaid. Medicaid.gov. November 10, 2025. Accessed January 14, 2026. https://www.medicaid.gov/medicaid/prescription-drugs/medicaid-drug-rebate-program

5. Medicaid National Drug Rebate Agreement (NDRA) | Medicaid. Medicaid.gov. December 29, 2022. Accessed January 14, 2026. https://www.medicaid.gov/medicaid/prescription-drugs/medicaid-drug-rebate-program/medicaid-national-drug-rebate-agreement-ndra

6. Federal Medical Assistance Percentage (FMAP) for Medicaid and Multiplier. Accessed January 19, 2026. https://www.kff.org/medicaid/state-indicator/federal-matching-rate-and-multiplier/?currentTimeframe=2&sortModel=%7B%22colId%22:%22FMAP%20Percentage%22,%22sort%22:%22desc%22%7D#

7. Levinson DR. States' Collection of Offset and Supplemental Medicaid Rebates (OEI-03-12-00520; 12/14). Published online December 2014. https://oig.hhs.gov/oei/reports/oei-03-12-00520.pdf

8. *42 U.S.C. § 1396b(m)(2)(A)(Xiii)*.; 2026. Accessed January 13, 2026. https://uscode.house.gov/view.xhtml?req=(title:42%20section:1396b%20edition:prelim)

9. Mooney DeBoy A. *Changes to Calculation of Average Manufacturer Price (AMP) under Medicaid Drug Rebate Program for Authorized Generics (as Required under the Pub. L. 116-59, the Continuing Appropriations Act, 2020, and Health Extenders Act of 2019)*. Centers for Medicare & Medicaid Services; 2019. Accessed January 13, 2026. https://www.medicaid.gov/medicaid-chip-program-information/by-topics/prescription-drugs/downloads/rx-releases/mfr-releases/mfr-rel-111.pdf

10. Further Recommendations on Calculation of Average Manufacturer Price (AMP) of Brand Name Drug and Authorized Generic Drugs (resulting from statutory changes in Continuing Appropriations Act, 2020, and Health Extenders Act of 2019). Medicaid Drug Rebate Program Notice: Release No. 112. https://www.medicaid.gov/prescription-drugs/downloads/mfr-rel-112.pdf

© 2026 El-Kilani ZS et al. *JAMA Health Forum*

11. Manufacturer Responses to the Medicaid Drug Rebate Cap Removal. Office of Inspector General | Government Oversight | U.S. Department of Health and Human Services. December 18, 2025. Accessed January 14, 2026. https://oig.hhs.gov/reports/work-plan/browse-work-plan-projects/srs-e-26-003/

12. National Average Drug Acquisition Cost | Medicaid. Accessed January 19, 2026. https://www.medicaid.gov/medicaid/nadac

13. Pharmaceutical Supply Chain Intermediary Margins in the Retail Channel. ASPE. January 14, 2025. Accessed January 19, 2026. http://aspe.hhs.gov/reports/margins-retail-channel

14. Medicare Drug Price Negotiation Program: Negotiated Prices for Initial Price Applicability Year 2026 | CMS. Accessed January 19, 2026. https://www.cms.gov/newsroom/fact-sheets/medicare-drug-price-negotiation-program-negotiated-prices-initial-price-applicability-year-2026

15. Hernandez I, Wouters OJ, Cousin EM, Kirihennedige AS, Sullivan SD. Interpreting The First Round Of Maximum Fair Prices Negotiated By Medicare For Drugs. *Health Affairs Forefront*. doi:10.1377/forefront.20240830.863408

16. Gifford K, Lashbrook A, Payne C. *State Approaches to Managing the Medicaid Pharmacy Benefit: Insights from a National Survey for State Fiscal Years 2023 and 2024*. Health Management Associates (HMA); 2024. https://www.healthmanagement.com/wp-content/uploads/2024-Medicaid-Rx-Survey-Rpt_FINAL.pdf

© 2026 El-Kilani ZS et al. *JAMA Health Forum*
