# Supplementary material for: Authorized Generics as a Manufacturer Response to the Medicaid Rebate Cap Removal
Source: JAMA Health Forum. 2026 May 8;7(5):e260680. doi: 10.1001/jamahealthforum.2026.0680 (PMC13156780; doi:10.1001/jamahealthforum.2026.0680)
Supplement: Supplement 2. — Data Sharing Statement [file jamahealthforum-e260680-s002.pdf]

Data Sharing Statement

El-Kilani. Authorized Generics as a Manufacturer Response to the Medicaid Rebate Cap Removal. *JAMA Health Forum*. Published May 08, 2026.
doi:10.1001/jamahealthforum.2026.0680

Data
Data available: No

Additional Information
Explanation for why data not available: We are willing to make the code available which leverages public data as well as additional material mentioned in appendix.
